# Green-Synthesized Nanomaterials for Kidney Cancer: Current Progress and Future Perspectives

**DOI:** 10.3390/ijms27188113

**Published:** 2026-09-11

**Authors:** Mariam R. Khalifa, Doaa S. R. Khafaga, Marwa T. Abo Gabal, Marwa Mohamed Abd El-Monem, Sara S. Zeidan, Shimaa S. Attia, Safaa Mahmoud Mohamed Abdelkhalek

**Affiliations:** 1 Biochemistry Department, Faculty of Science, Ain Shams University, Cairo 11566, Egypt; mariamraafat@sci.asu.edu.eg (M.R.K.); marwataha134@gmail.com (M.T.A.G.); marwamohamed@sci.asu.edu.eg (M.M.A.E.-M.); sarasamir0105128@sci.asu.edu.eg (S.S.Z.); 2 Department of Basic Medical Sciences, Faculty of Medicine, Galala University, New Galala City, Suez 43511, Egypt; 3 Anatomy & Embryology Department, Faculty of Medicine, Ain-Shams University, Abaseya, Cairo 11566, Egypt; dr.shimaa.s@gmu.ac.ae; 4 Biomedical Sciences Department, College of Medicine, Gulf Medical University, Ajman 4184, United Arab Emirates; 5 Department of Pathology, Faculty of Medicine, Ain Shams University, Abaseya, Cairo 11566, Egypt

**Keywords:** kidney cancer, renal cell carcinoma, drug delivery, green synthesis, nanoparticles

## Abstract

The kidney is an essential organ that has a crucial role in preserving homeostasis within the human body. Kidney cancer is considered a major clinical challenge owing to its resistance to traditional treatments such as chemotherapy, radiotherapy, and immunotherapy. Nanoparticles (NPs) gain great attention in cancer therapy due to their low toxicity, biocompatibility, and targeted drug delivery capability. This review focuses on the current role of green-synthesized NPs in renal cell carcinoma management and their applications in targeted drug delivery and cancer-specific targeting mechanisms with the demonstration of the environmentally friendly green synthesis approaches utilizing biological resources such as plant extracts and microorganisms, highlighting their advantages over conventional synthesis methods in terms of biocompatibility, sustainability, and reduced toxicity. Moreover, the therapeutic potential of nanomaterials is discussed, including magnetic NP-mediated thermal therapy, photothermal therapy, gene delivery systems, and RNA interference-based strategies. We underscore the challenges and limitations of NPs, including in vivo toxicity, biodistribution, clinical translation, large-scale production, batch-to-batch variability, long-term safety, scalability, repeatability, regulation, and reproducibility. There is growing potential to improve the treatment of kidney cancer. Hence, the promising prospects of nanotechnology in kidney cancer treatment provide a foundation for future research and clinical application. This comprehensive article highlights the significant contributions of nanomedicine to oncology and shows an optimistic perspective for more effective and precise treatment strategies for kidney cancer.

## 1. Introduction

The kidney plays a vital role in maintaining electrolyte balance and body fluids via reabsorption, removing waste metabolites from the bloodstream via secretion, and regulating blood pressure through the renin–angiotensin–aldosterone system, all of which are essential for survival. However, kidney cancers pose a significant risk to human health and life [1,2]. Renal cell carcinoma (RCC) is a heterogeneous group of cancers that accounts for approximately 80 to 90% of renal malignancies and originates in the tubular epithelium. Clear cell RCC (ccRCC), papillary RCC, and chromophobe RCC are three major histological subtypes of RCC. Other less common types of kidney tumor, which account for about 15%, include 8% transitional cell carcinoma, 5–6% Wilms’ tumor or nephroblastoma, (<1%) collecting duct tumors, (<1%) renal medullary sarcoma, and (<1%) renal sarcoma [2]. It is demonstrated that Trichloroethylene is labeled as a carcinogenic compound to humans by the International Agency for Cancer Research. Occupational exposure to it relates to renal cancer [3]. Age, race, and gender are factors that contribute to RCC development. In 2023, an estimated 81,800 new cases of RCC were diagnosed in the U.S., ranking it as the ninth most common cancer among females and the sixth most prevalent malignancy in men [4,5]. It occurs at twice the rate in men compared to women [6]. It is considered the third most common urologic malignancy, impacting over 400 thousand individuals annually [7]. In RCC, there are multiple risk factors, including both genetic and acquired risk factors. Hypertension, analgesic use, obesity, smoking, diabetes, and chronic kidney diseases (CKDs) are the most common associated or acquired risk factors for RCC [8,9].

For genetic factors, research on familial RCC has identified mutations in at least 11 genes, including *BAP1*, *FH*, *MET*, *FLCN*, *SDHB*, *SDHC*, *SDHD*, *TSC1*, *PTEN*, *TSC2*, and *VHL*. Some of these genetic mutations have also been linked to the development of sporadic RCC [10]. The two common genes included in the pathogenesis of RCC are the Von Hippel–Lindau (*VHL*) gene and the protein polybromo-1 (*PBRM1*) gene [11]. An autosomal-dominant mutation in the *VHL* gene leads to *VHL* disease, which triggers the activation of vascular endothelial growth factor (VEGF), angiogenesis, and hypoxia pathways [12] and consequently leads to the development of RCC; this is the most common syndrome that confers a greater risk of RCC developing [13,14]. The most important prognostic determinants of 5-year survival are the tumor stage, the grade, the local extent of the tumor, the presence of regional nodal metastases, and evidence of metastatic disease at presentation. The primary sites of metastasis for RCC include the lungs, bones, liver, lymph nodes, adrenal glands, and brain [14,15,16]. The pancreas, breast, skin, thyroid, and muscle are also atypical sites in which RCC metastasizes [17,18].

Treatment approaches for RCC limited to the kidney involve surgical resection through partial or radical nephrectomy or removal of the kidney; ablative techniques by which heat or cold causes the removal of the malignant tissue such as cryo-, radio-, and microwave ablation and radiation; or active surveillance, which means regular radiographic studies to track tumor growth over time, particularly for patients with renal masses smaller than 2 cm. Partial nephrectomy offers a cancer-specific survival rate exceeding 94% over five years for patients with renal masses smaller than 4 cm [19]. Patients with early-stage RCC can be cured by surgical resection. However, some of them may develop recurrent or metastatic disease at diagnosis, so systemic treatment is required, for example with sunitinib, pazopanib, sorafenib, and axitinib, which belong to the tyrosine kinase inhibitor (TKI) class. Although RCC is intrinsically radioresistant, radiation therapy is one of the treatment approaches for RCC [20,21,22]. For metastatic RCC, the agents approved for treatment include anti-vascular VEGF monoclonal antibodies (mAbs) such as bevacizumab. Immunotherapy with checkpoint inhibitors that target the interactions between cancer immune cells is becoming prominent, for example a combination of anti-programmed death-1/programmed death ligand-1 (anti–PD-1/PD-L1) and a cytotoxic T-lymphocyte-associated protein 4 (CTLA-4) inhibitor, such as ipilimumab plus nivolumab. Mammalian target of rapamycin (mTOR) inhibitors such as temsirolimus and everolimus are also used. The combination of tyrosine kinase inhibitors with immune checkpoint inhibitors has also been explored. The combination of checkpoint inhibition and VEGF inhibition, such as bevacizumab plus atezolizumab and nivolumab plus ipilimumab plus cabozantinib, is also used. With those combinational therapies, the rates of tumor response were from 42% to 71%; additionally, median overall survival was from 46 to 56 months [19,23,24,25]. Surgery, chemotherapy, and radiation therapy are three major approaches for kidney cancer treatment; however, one of the most alarming consequences is the reduced effectiveness of chemotherapy and radiation therapy. A significant recurrence rate of as much as 40% after surgery is also one of the drawbacks of this conventional therapy. Although immunotherapy and targeted therapy have had novel approaches during the past decades, severe side effects, high prices, and the complex nature of molecular targets represent limitations of these therapies [26,27,28]. The development of resistance to cancer therapy is a significant problem for RCC patients. Most resistance mechanisms, like target gene changes or bypass pathway activation, are biological. Pharmacologic resistance occurs through poor drug penetration or cellular pumps expelling the drug. Some involve phenotypic shifts, such as epithelial–mesenchymal transition (EMT) [29,30,31].

Currently, nanotechnology is attracting increasing interest in its application to cancer because it has unique advantages for imaging and diagnosis, drug delivery, and the medicinal properties of some nanomaterials themselves. Nanomaterials possess prospective use in non-invasive diagnostics, nerve injury treatment, and customized nano-drug therapy for renal diseases [32,33]. Over the past five years, several studies have explored the use of NPs for kidney cancer treatment. When compared to traditional therapies, nano-drug delivery allows for targeted and prolonged release of therapeutics while overcoming the drawbacks of low solubility, poor absorption, and low accumulation in target organs. The use of biocompatible carriers, such as liposomes, dendrimer polymers, hydrogels, micelles, lysozymes, adenoviruses, and elastin-like polypeptides, is a prerequisite for nanomedicine in the treatment of kidney diseases. They enhance the efficacy of the drugs they carry due to their accurate renal uptake, longer half-life, and targeted organ distribution. Size, shape, surface parameters, hydrophobicity, and other properties determine the application potential of carriers [34,35]. Numerous studies and clinical trials have produced successful nanomedicines for the systemic management of kidney cancers, encompassing both diagnosis and therapy [27]. NPs characterized by dimensions between 1 and 100 nm have gained great attention in recent years owing to the distinctive physicochemical features that distinguish them from their bulk materials. Enhanced reactivity, increased surface area, and quantum effects are unique properties of NPs that make them employed in imaging, diagnostics, and drug delivery systems with lower side effects [36,37]. NPs can overcome chemo-drug or radiotherapy resistance mechanisms and minimize cancer-therapy-related side effects [38]. The development of nanomaterials in a variety of fields in recent years highlights their essential role as one of the key components in building an effective treatment platform against RCC as drug drivers [39]. Engineered NPs have significantly increased cancer treatment by encapsulating and delivering therapeutic and diagnostic substances that would normally be limited by poor pharmacokinetics and toxicity to target tumor cells. In order to maximize efficacy and further reduce off-target toxicities, nanomedicine has expanded to target key tissues and cells implicated in carcinogenesis, such as immune and stromal cells, by utilizing our growing understanding of the tumor microenvironment [40].

Biosynthesis is known as the process of generating complex molecules via biological systems, particularly in the context of NP, in which they can be synthesized with the use of living organisms such as fungi, bacteria, and plants, as well as byproducts of their metabolism. Traditional chemical and physical methods of NP synthesis possess several limitations, such as the generation of hazardous waste, the consumption of high energy, and the use of toxic reagents, while the green or biosynthesis approach is characterized by its reliable, maintainable, and eco-favorable synthesis and its inhibition of unsafe or undesired byproducts [41,42,43].

Inorganic NPs synthesized through green approaches using derivatives of antioxidants found in medicinal plants, such as flavonoids and polyphenols, show promise as a potential treatment. In addition to avoiding the use of hazardous chemical reagents, this method enables the development of NPs with particular physicochemical characteristics, such as size, charge, and shape, that make it easier for them to pass through the digestive system, avoid the immune system, and be delivered specifically to renal tissue [44]. This environmentally friendly method frequently produces biocompatible NPs for biomedical use using less hazardous chemicals that have a lower impact on the environment. Utilizing the full benefit of natural sources, green synthesis offers a viable, cost-effective, environmentally friendly, and scalable alternative to traditional chemical approaches, particularly in targeted cancer therapy [45]. Inorganic NPs, which include metals and metal oxides (e.g., zinc oxide, gold, silver, iron oxide, gadolinium, and titanium dioxide particles), show great promise as medical tools for imaging cells, biosensing, delivering drugs and genes, and treating cancer [46]. Metal nanoparticle (MtNP) synthesis with the use of microorganisms and plants is known as an efficient and environmentally friendly approach for the enhanced utilization of microorganisms [47]. Anti-cancer activity, magnetically responsive drug delivery, photothermal therapy, and bioimaging are applications of NPs synthesized by green synthesis [48]. Because of different reductase enzymes, several microorganisms can act by collecting and detoxifying heavy metals. These microbes convert metal precursors, especially metal salts, to MtNPs. The microbial method attracts scientists and researchers due to microorganisms’ ability to withstand extreme conditions, including variable pH, temperatures, and pressures [49]. MtNPs can be produced intracellularly or extracellularly by a wide range of microorganisms, including bacteria, fungi, yeasts, and microalgae. These microbes can make organic compounds within their cells and then move them outside to other parts of their bodies [50,51]. Additionally, the unique features of microbial cells enhance NP synthesis by enabling them to act as reducing and capping agents, thereby offering safe use, rapid growth, and easy maintenance [52]. Also, NP synthesis using plant extracts promotes the minimization of environmental effects and decreases costs associated with complex instrumentation and toxic chemicals. Plant extracts are promising agents for NPs synthesis because they are rich in phytochemicals such as phenolics, terpenoids, proteins, alkaloids, and flavonoids, which can reduce metal ions and stabilize the resulting NP [53,54,55].

Green chemistry and nanotechnology have expanded MtNPs that can safely interact with living creatures. Plant-based NP synthesis is simple, safe, cost-effective, and large-scale compared to previous approaches. Eco-friendly methods can manufacture well-defined NPs that have precise sizes and shapes suitable for commercial and biomedical applications [56].

Lipid-based nanoparticles (LNPs), including liposomes, micelles, nanoemulsions, solid lipid NPs, and nanostructured lipid carriers, are advanced drug delivery technologies that provide unique approaches to the diagnosis and treatment of RCC. The exceptional biocompatibility, biodegradability, prolonged circulation, and tumor-targeting properties of these LNPs allow for precise drug delivery, controlled release, and evasion of drug resistance mechanisms [57].

NPs are particularly significant in renal applications due to their ability to deliver drugs to affected tissues and the need for their minimal adverse effects compared to traditional therapies. As a potential game-changer in the targeted diagnosis and treatment of renal disorders, the size of NPs affects their ability to penetrate the kidney through glomerular filtration or tubular secretion pathways [58]. However, renal interactions are also influenced by surface charge, shape, and functionalization. Particles smaller than ~5–6 nm are rapidly cleared via glomerular filtration [59], whereas particles near the filtration threshold may exhibit size-dependent interactions at the glomerular interface and tubular handling depending on physicochemical properties [60]. At ultrasmall scales, atypical interactions with the glomerular glycocalyx have been reported in specific nanocluster systems [61]. In contrast, particles above the filtration threshold (~6–8 nm) exhibit markedly reduced renal clearance and may show localization within glomerular structures [62]. Systemic clearance via the reticuloendothelial system becomes increasingly dominant with increasing particle size [63]. Overall, renal NP behavior cannot be fully described by a single size cutoff but instead reflects a combination of physicochemical and biological factors.

## 2. Limitations of Traditional Drugs for RCC

Chemotherapy, cytokines, TKIs, and immune checkpoint inhibitors are common treatments used for RCC, as shown in Table 1. Due to the high risk of multidrug resistance (MDR), selective delivery of drugs, off-target effects, and severe toxicity of targeted chemotherapeutics, their application for RCC is limited, and treatment plans are hindered. One of the promising new approaches for treating cancer is the combination of chemotherapy and immunotherapy. Although the combination of pembrolizumab and axitinib achieves an objective response, it causes side effects such as acute kidney injury, pulmonary embolisms, pneumonitis, and abnormal liver function. Additionally, higher doses of these drugs are required to increase their bioavailability, but this can cause high toxicity to normal cells and MDR development [19,64].

## 3. Effective Role of Various Nanomaterials for Kidney Cancer

Nano-drug conjugates offer a more accurate approach for drug delivery, enhancing therapy effectiveness while lowering systemic toxicity. Traditional treatments, such as chemotherapy, surgery, and radiation, can cause nonspecific toxicity and adverse side effects [68]. Nanomedicine provides many technological advances for kidney cancer management. Using active or passive targeting, agents or drugs can be loaded onto NPs and guided to certain cell types within target organs, allowing them to pass through the body’s biological barriers. They can be altered to ensure appropriate circulation times and can contain two or more therapeutic drugs for synergistic effects. Diagnostic assays for kidney cancer that depend on nano-scale sensors allow biomarker detection at the femtomolar level. Furthermore, they can overcome mechanisms of resistance to chemotherapy or radiation [69]. Nanomedicine may be able to reduce the negative effects of cancer therapy, allowing for controlled drug release and improving the precision of treatment [70].

Gold nanoparticles (AuNPs) have several benefits for cancer treatment. Their size modification, low toxicity, and biocompatibility make them perfect for drug delivery systems. AuNPs are very useful for therapeutic applications like photothermal therapy and radiosensitization, as well as diagnostic imaging, because of their strong optical and physical characteristics. Additionally, the functionalization of AuNPs with different targeting ligands increases their selectivity, and their precise delivery to cancer cells is made possible, reducing harm to healthy organs. They are more efficient in cancer treatment due to their capability to function as both therapeutic and diagnostic agents, “theranostics”. AuNPs’ surface modification capabilities have been used to deliver a variety of compounds, like proteins, medications, and nucleic acids. Additionally, the strong localized surface plasmon resonance (LSPR) characteristics of AuNPs make them suitable for surface-enhanced Raman scattering, which enables accurate and effective biomolecule identification and detection [71].

AuNPs could be used to deliver genetic materials and anticancer drugs in combination with chemotherapeutic drugs for synergistic effects. Furthermore, AuNPs may enhance apoptosis and the production of reactive oxygen species (ROS) by increasing chemosensitivity. AuNPs may increase the cytotoxicity of chemotherapeutic drugs against tumor cells in a photothermal therapy manner. Drug release at the site of the tumor can be triggered using pH, redox, and photosensitive AuNPs. The ability of AuNPs to penetrate cell membranes may be further enhanced by modifications to their surfaces that increase stability, reduce NP aggregation, and improve adhesion to target cells and anticancer drugs. By inducing a longer release with low concentrations of chemotherapeutic agents while sustaining their anticancer efficiency, AuNPs can protect tumor cells from drug resistance [72]. Biosynthesized CWAuNPs, green-synthesized AuNPs from *Curcuma wenyujin*, are effective anticancer agents that cause apoptosis in the A498 renal cell carcinoma cell line [73].

Silver nanoparticles (AgNPs) show anticancer, anti-inflammatory, and anti-angiogenic properties [74]. Silver ions promote ROS production, which is thought to be the primary cause of most cell deaths by deactivating the production of ATP and DNA replication [75]. AgNPs and their conjugates with anti-tumor agents induce oxidative stress, ROS generation, DNA damage, the arrest of the cell cycle, and tumor cell death by apoptotic and nonapoptotic pathways [76]. AgNPs stimulate necroptosis pathways by activating proteins, including RIP-1, RIP-3, and MLKL, which make membrane pores, leading to calcium influx and cell death [77]. Biogenic AgNPs have anticancer properties that cause cancer cells apoptosis via mitochondria-dependent and caspase-dependent pathways. The cellular uptake of bAgNPs may occur by endocytosis, phagocytosis, and pinocytosis; then, AgNPs stimulate the generation of ROS inside the cells, causing the activation of p53 proteins and caspase-3 sequences. Caspase-3 causes the apoptosis of cells by arresting the G2/M phase of the cell cycle [78,79]. AgNPs synthesized from *Malva sylvestris* had cytotoxicity activity against RCC cell lines [80].

Iron oxide nanoparticles (IONPs) have various specific magnetic characteristics, including superparamagnetic properties and high magnetic perceptivity, known for their biocompatibility and low toxicity [81]. The design of IONPs has an important role in the determination of biocompatibility and efficiency. IONPs are great contrast agents for MRI because of their special size and modifications. The size and biodistribution are affected by several modifications, including surface coating and attachment to biomolecules, which ultimately facilitate target tissue accumulation and effective imaging [82].

IONPs aid in both accurate diagnosis and real-time monitoring of treatment response. IONPs can also be used in hyperthermia for cancer therapy. Light-activated IONPs produce heat within the microenvironment of the tumor, resulting in the direct destruction of cancer cells [83]. Hyperthermia is able to inhibit the proliferation, invasion, and migration of RCC cells; moreover, it promotes cell apoptosis. Hyperthermia can develop the immunogenicity of RCC cells by stimulating immunogenic cell death [84].

Carbon nanomaterials (CNs) are carbon-based compounds with optical, mechanical, and electrical properties, so they could be used in the medical field [85]. These properties make them effective carriers for biological molecules and drug delivery because of their surface area and biocompatibility [86]. CNs have great potential in gene, peptide, and drug delivery for RCC treatment. They also have roles in hyperthermia, photodynamic therapy, and photoacoustic therapy; the interaction of CNs with the tumor microenvironment (TME) is estimated, as they can modulate the immune response and regulate tumor hypoxia [87].

Carbon nanotubes (CNTs), graphene, and fullerenes are examples of CNs used in drug delivery to different specific targets [88]. A fullerene is an allotropic form of carbon with a hollow sphere, an ellipsoid, and a tube shape, and it comes in many other sizes [89]. Fullerene derivatives have anti-tumor characteristics, including anti-oxidation, immunological activation, cell cycle arrest, anti-metastasis, tumor angiogenesis suppression, and drug resistance inhibition [90]. Fullerenes accumulate within the kidney cells, pass through the urine, and exhibit anti-oxidative stress in tumors. Fullerenol C_60_(OH)_24_ has great potential in anti-cancer treatment as a ROS-scavenger by protecting kidney cells and other primary organs from doxorubicin toxicity [87]. CNTs have characteristics such as biocompatibility, good stability, immunogenic suppression, ease of size modification, and high drug-loading capacity, so could be used in the classical treatments of cancer or conjugation with chemotherapeutics [91]. CNTs have advantages, including a small size, an ultra-high aspect ratio, surface modification capabilitiy, and the high capacity to encapsulate drugs, making them unique materials for nano-drug delivery approaches. The high aspect ratio of CNTs gives them the ability to penetrate cell membranes efficiently and accumulate within them [92]. CNTs can also be used in phototherapy due to their optical properties [93]. CNTs can be classified according to how many layers of graphene are in the cylindrical tube [94]. CNTs are classified into single-walled (SWCNTs) or multi-walled (MWCNTs) forms. MWCNTs have a number of SWCNTs layered inside one another [95]. MWCNTs can produce heat when exposed to near-infrared (NIR) radiation, resulting in the thermal ablation of kidney tumors [96]. 

Graphene-based nanomaterials (GBNMs) are classic two-dimensional (2D) nanocomposites with physicochemical characteristics and unique structures exhibiting high potential in cancer therapy, including photothermal therapy (PTT), photodynamic therapy (PDT), and combination therapy [97]. Graphene and graphene oxide NPs have a high surface area, a high drug loading capacity, ease of functionalization, and ROS generation capabilitiy, making them suitable for the transport of anticancer genes, diagnostic agents, and drugs to targeted tissue [98]. NIR light is absorbed by GBNMs, particularly reduced graphene oxide (rGO) and graphene oxide (GO), which produces heat that is helpful in PTT of cancer. Despite having structural similarities, GO and rGO have different anticancer therapy effects because of their varied photothermal capacity [99]. Graphene’s surface could be non-covalently and covalently modified with functional groups and anti-cancer drugs to effectively target cancer cells [100]. Fe single-atom-decorated graphene oxide (Fe1-GO) induces autophagy in RCC and reduces tumor weight significantly with a suitable cytotoxic effect on normal cells, exhibiting high potential in kidney cancer therapy in addition to the modulation of ROS [101].

Liposomes are vesicles composed of a hydrophilic water core and a hydrophobic lipid bilayer with amphiphilic phospholipids, including phosphatidylserine, phosphatidylcholine, phosphatidylglycerol, and phosphatidylethanolamine. They can encapsulate hydrophilic and hydrophobic molecules. They can be used effectively in gene delivery and RCC-targeted drug delivery therapy [102], as shown in Table 2. In a previous study, liposomes were encapsulated with a multi-receptor TKI (XL184) to evaluate its therapeutic potential, showing that the cMet pathway, which is involved in VEGF therapy resistance and RCC development, was inhibited [103]. Sorafenib is a TKI drug that has clinical potential for the treatment of advanced RCC. Magnetic solid LNPs are loaded with sorafenib to enhance drug delivery to tumors with low side effects on healthy cells in the remote magnetic field [104]. Another study shows that the tumor-targeted liposomes loaded with everolimus and vinorelbine improve RCC treatment [105].

Dendrimers are NPs with highly branched and tree-like structures. They have high drug-loading capacity and ease of modification of their size and surface with many functional groups on their surface for the attachment of imaging agents and the targeting of ligands to them [106]. Dendrimers are polymeric macromolecules. They have unique physicochemical properties, intramolecular cavities, and easy surface functionalization. Due to these structures, they can be used as anticancer agents. Dendrimers can deliver DNA, RNA, and drugs to cancer cells with no side effects on healthy cells [107,108]. Dendrimers have the ability to control anticancer agent release in the tumor microenvironment. They can also combine multiple therapeutic strategies, such as photothermal and photodynamic therapies, to exhibit anticancer effects [109].

Micelles are self-assembling NPs with a hydrophobic core and hydrophilic shell [110]. In cancer gene therapy, the micelles can offer a platform for the co-delivery of RNAi and non-coding RNAs. Micelles can deliver anti-tumor agents, both natural and synthesized. Additionally, synergistic cancer therapy is mediated by the co-delivery of genes and drugs. A variety of smart micelle types, such as multi-sensitive micellar nanostructures, pH-, light-, and redox-sensitive micellar nanostructures, may be beneficial in targeted cancer treatment [111]. Polymer micelles can effectively encapsulate hydrophobic agents within their hydrophobic core, leading to an increase in the water solubility of hydrophobic drugs, elimination of drug-induced side effects, control of drug release rate, and protection of drug molecules from degradation by specific media (pH and temperature) [112]. Peptide amphiphile micelles (PAMs) have been utilized to target the kidneys, particularly providing peptide ligands that bind to renal receptor megalin, ensuring higher accumulation in kidney tissues [113].

The comparison highlights that no single nanocarrier platform is universally superior for all therapeutic applications. Liposomes offer excellent biocompatibility and versatility for drug encapsulation, dendrimers provide extensive surface functionality and high loading capacity, whereas micelles are particularly effective for the delivery of hydrophobic agents and can be engineered for kidney-specific targeting. Therefore, the selection of an appropriate carrier should be guided by the therapeutic objective, target renal compartment, and desired pharmacokinetic profile.

## 4. Nanomaterials’ Targeting Mechanism for Cancer Cell Death

Large and small NPs have different mechanisms for entering the cell. Small NPs interact with the cell membrane and could enter the cells through caveolin receptor-mediated uptake. For large NPs, clathrin receptor-mediated uptake or endocytosis is preferred. Aggregated NPs enter the cell via phagocytic pathways, while non-aggregated NPs commonly enter through non-phagocytic pathways. Before entering the cytosol, the NPs go through surface modifications or enter the endosome–lysosome complex. Alternatively, the NPs enter the cell directly into the cytosol and interact with the cytosolic proteins [114]. After interacting with ROS, the NPs often release metal ions into the cell, causing the cell to produce more ROS. Metal ions readily attach to SH groups in proteins, rupturing S-S bridges and altering cell physiology [115]. Different programs for cell death have been activated after all of the previous processes [116].

Apoptosis, or programmed cell death, occurs through two primary pathways: extrinsic and intrinsic. The extrinsic pathway can be categorized into three mechanisms: (a) activation of death receptors, leading to the caspase-8/3 cascade; (b) death receptor activation, which triggers caspase-8, BID cleavage, mitochondrial outer membrane permeabilization (MOMP), and subsequently the caspase-9/3 cascade; and (c) dependence receptor activation due to ligand deprivation, which then initiates caspase-9/3 activation either directly or via MOMP.

Conversely, intrinsic apoptosis is initiated by intracellular stress and can follow either caspase-dependent or caspase-independent routes. Nanomaterials have been shown to induce apoptosis through two biological pathways. In the intrinsic pathway, ROS accumulation leads to mitochondrial membrane depolarization, causing the release of cytochrome c into the cytosol. This release enables cytochrome c to interact with apoptotic protease-activating factor-1 (Apaf-1) and procaspase-9, triggering the caspase-9/3 cascade. The activation of caspase-3, an executioner caspase, leads to the proteolytic cleavage of multiple cellular proteins, such as poly ADP-ribose polymerase-1 (PARP-1).

The extrinsic pathway is also influenced by ROS, which enhances the expression of Fas, a crucial death receptor. This, in turn, stimulates the caspase-8/3 cascade and modifies various pro-apoptotic proteins through dephosphorylation and post-translational modifications. Key proteins involved in this process include Bcl-2-like protein 11 (Bim), Bcl-2 homologous antagonist killer (Bak), Bcl-2-associated X protein (Bax), Bcl-2-associated death promoter (Bad), BH3-interacting domain death agonist (Bid), harakiri (Hrk), and other B-cell malignant lymphoma family proteins. The activation of the pro-apoptotic protein Bcl-Xs prompts its translocation to the mitochondria, where it serves as a signal to initiate the apoptotic process [117].

Caspase-3, an executioner caspase, activates caspase-activated DNase (CAD), leading to the targeted fragmentation of DNA. The resulting DNA damage subsequently triggers the activation of the ATM kinase and JNK-mediated signaling pathways, which, through phosphorylation, enhance the activity of p53. Additionally, JNK facilitates cytochrome c release via a Bid-Bax-dependent mechanism. This cascade further activates several pro-apoptotic regulators, including nuclear factor kappa-light-chain-enhancer of activated B cells (NF-κB), p53 upregulated modulator of apoptosis (PUMA), BAX, transformation-related protein 63 (p63), and transformation-related protein 73 (p73), ultimately driving apoptosis. Moreover, JNK plays a role in promoting the release of the second mitochondrial-derived activator of caspases (Smac)/direct IAP-binding protein with low pI (Diablo), which counteracts caspase-8 inhibition, thereby facilitating caspase activation [118].

Autophagy represents a controlled biological process of programmed cell death controlled by autophagy-related genes (ATGs). It is triggered by various extracellular or intracellular stressors. Originally serving as a protective mechanism to enhance cell survival, autophagy can become harmful when excessively activated, leading to cell death through its cytotoxic effects.

Beclin-1, along with its binding partner myristoylated serine kinase Vps15/p150, forms the Beclin-1 interactome. In cytoprotective autophagy, this interactome regulates the activity of Class III phosphatidylinositol 3-kinase (PI3KCIII), which results in the suppression of Beclin-1’s tumor-suppressive function. Autophagy contributes to tumor suppression by enhancing the expression of pro-death proteins such as Beclin-1, p53, Class III and I PI3K, and mammalian target of rapamycin complexes 1 and 2 (mTORC1/C2), while simultaneously reducing the levels of pro-survival proteins like Bcl-2 and B-cell lymphoma extra-large (Bcl-XL) [119].

p53 has an essential role in autophagy by facilitating the transformation of microtubule-associated protein 1A/1B-light chain 3 (LC3) from its LC3-I form to LC3-II, thereby initiating the autophagic process. The UV irradiation resistance-associated gene (UVRAG) protein binds to Beclin-1, while protein phosphatase 1-binding protein Bifocal (Bif-1) interacts with Beclin-1 via UVRAG, both serving as positive regulators that enhance PI3KCIII lipid kinase activity and promote autophagy [114].

Nanomaterials can trigger autophagy through multiple mechanisms, including oxidative stress, the accumulation of damaged proteins leading to organelle stress, modifications in gene expression, and disruption of kinase-mediated signaling pathways [120]. The accumulation of autophagic vacuoles in response to nanomaterials is considered an adaptive mechanism by cells. Studies have shown that various types of nanomaterials can increase autophagic vacuole formation, as observed in human cells, animal models, and in vivo studies. Within the autophagosome, NPs undergo degradation before being released into the cytoplasm [121].

Programmed necrosis is a distinct type of programmed cell death triggered by death ligands such as TNF-α, TNF-related apoptosis-inducing ligand (TRAIL), and Fas ligand (FasL) upon binding to their respective receptors. This receptor activation leads to the formation of a signaling complex comprising caspase-8, Fas-associated protein with death domain (FADD), and receptor-interacting protein kinases (RIP1 and RIP3). The interaction between RIP1 and RIP3 subsequently activates metabolic enzymes, resulting in elevated production of ROS, which serve as key mediators of necrotic cell death. Under stress conditions, additional necrotic regulators—including PARP1, calpains, RIP kinases, and PAR polymers—become activated, further promoting necrosis. Notably, nanomaterials have been found to induce ROS-dependent necrosis, where RIP1 and RIP3 contribute to mitochondrial dysfunction either directly or indirectly by increasing intracellular calcium levels and activating NADPH oxidase, ultimately enhancing ROS production and driving programmed necrosis [114].

When NPs aggregate, their surface oxidation occurs more readily due to their high surface energy, which makes them prone to oxidation. As aggregation progresses, the overall surface energy of the NPs increases even further, facilitating the oxidation process and leading to the formation of metal oxide NPs. Metal oxide NPs undergo dissolution in aqueous media, leading to the release of metal ions [122].

## 5. Green Synthesis of Nanomaterials

**Figure 1:**
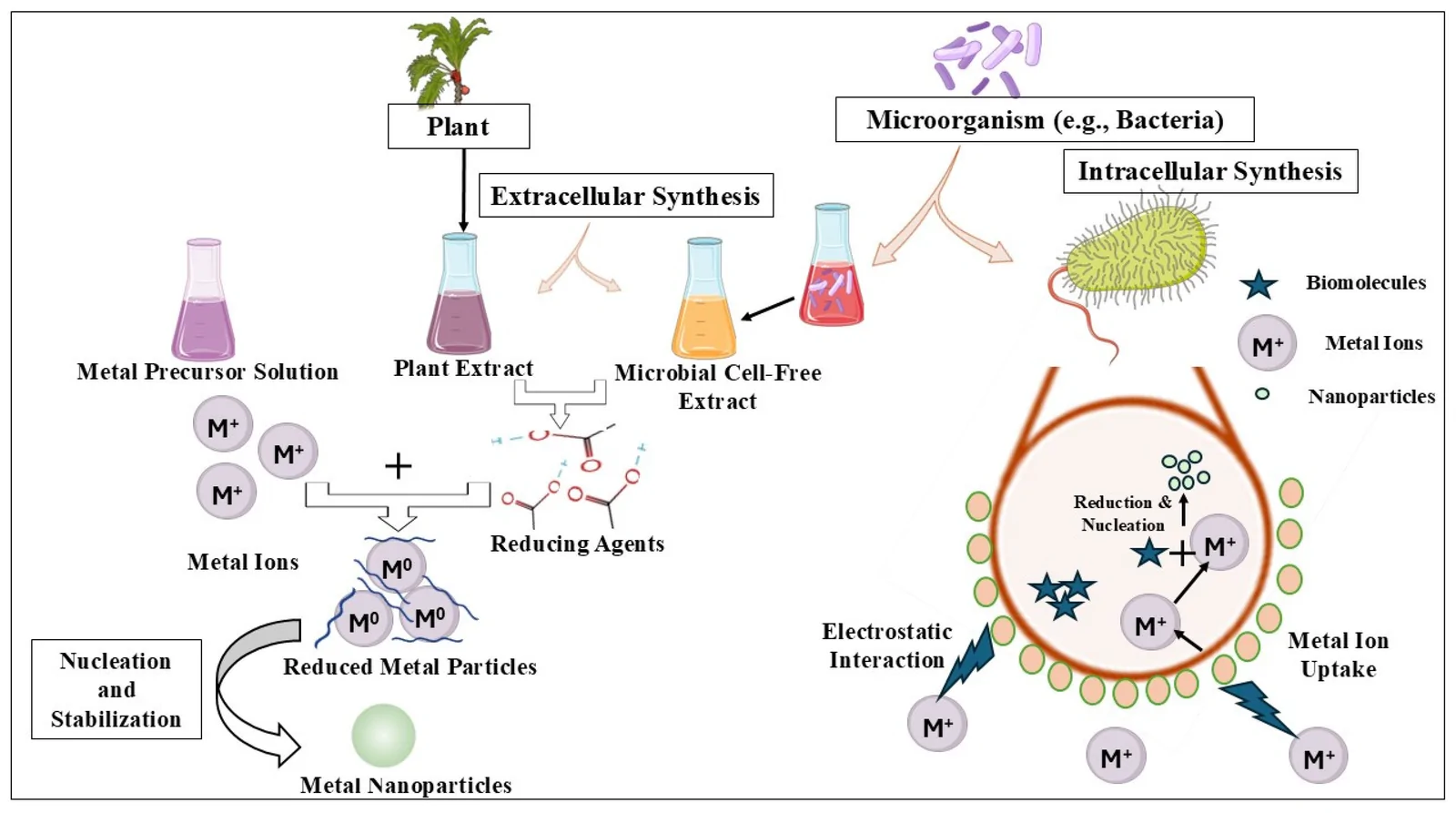
Green synthesis mechanism of MtNPs.

Toxic substrates are required in chemical processes in the synthesis of NPs, and this may consume a high amount of energy and have low productivity, generating harmful waste [123]. Instead of expensive physical or chemical methods, green synthesis is a bottom-up approach that uses living organisms, and their biomass is a cost-effective, clean, and less-toxic method for NP synthesis. In an environmentally friendly manner, biological systems such as plants and microorganisms are utilized for the green synthesis of NPs. These methods offer several advantages compared to traditional methods, involving lower toxicity, sustainability, and enhanced biocompatibility due to their dependence on the natural reductive properties of biological agents for metal ion reduction and NPs formation [124]. Metallic nano-therapeutics were created by biomolecules derived from plants (seed, root, stem, leaves, flowers, fruit, and peel) and microbes (fungus, bacteria, yeast, and algae) that are utilized as reducing agents and biocompatible coatings [125], as illustrated in Figure 1. Based on where NPs are formed, they are classified into intracellular or extracellular amalgamations. The synthesis of NPs within the living cells of plants or microorganisms represents the intracellular approach. The capability of plants to accumulate metals, which are then reduced to NPs in the cytosol due to the plants’ reductive potential [42,126]. The resulting NPs from intracellular synthesis are often uniform in shape and size because this process allows for NP synthesis in a controlled environment, as the biological system allows the regulation of particle growth. The second approach is extracellular synthesis, which involves the synthesis of NPs in the surrounding medium or outside the living cells. This method involves mixing biological extracts such as fungi or plant leaves with a solution of metal salt [127]. The extract contains a diverse range of biomolecules such as flavonoids, terpenoids, and polyphenols, which reduce metal ions to their zero-valent state by donating electrons to them. This reduction allows the formation of crystalline nuclei and subsequently their growth into NPs. Not only do these biomolecules act as reducing agents, but they also act as capping agents that help in the stabilization of NPs and the prevention of agglomeration to ensure the desirability and functionality of NPs [128,129]. Capping is essential for the chemical and physical properties of NPs, affecting their reactivity, solubility, and compatibility with biological systems [130]. Plants and microbes can be utilized for the creation of sustainable nanomaterials for the biomedical industry due to their easy availability, affordability, and biocompatibility. Owing to their accessibility, compositional distribution, safe handling, and occurrence of a wide range of metabolites, they have the potential for NPs production, which makes them serve as “biological laboratories” or “biological nanofactories”. Exposure time, pH, temperature, concentration of the medium, the type of reducing agent, concentration of the biological entity, and other variables determine the dimensions and characteristics of the NPs produced biologically. Also, capping and stabilizing agents affect NPs’ properties as they are required to reduce aggregation and regulate NPs’ growth. From metallic precursors, many nano-sized metals, metal oxides, metal sulfides, and metal phosphates can be created [131].

### 5.1. Plant-Based Synthesis

Plants contain secondary metabolites or bioactive compounds known as phytochemicals, which frequently have anti-inflammatory, antiviral, antifungal, antibacterial, and antioxidant effects. Plant parts such as seeds, fruits, stems, stalks, and bark contain a lot of naturally occurring substances, including flavonoids, saponins, polyphenols, terpenoids, flavones, alkaloids, tannins, and proteins, which enable them to create various nanomaterials and account for their extraordinary bio-reduction capability. During the synthesis of NPs, these compounds can act as reducing agents, stabilizers, and capping agents. Multiple phytochemicals or purified compounds are mixed with aqueous solutions containing metallic salts, even mono or polyvalent ions (e.g., metal nitrate or metal chloride). Metal ions in the solution are attracted to functional groups of phytochemicals [132]. Incubation after mixing is performed under light-controlled conditions to reduce metal salts and form MtNPs with their zero-valent ions [50,103]. For example, during the formation of AgNPs, ions of silver (Ag^+^) are reduced by phytochemicals to metallic silver (Ag^0^). The reduction process and formation of intermediate complexes facilitate the nucleation of metal atoms to produce small nuclei or clusters. This leads to the growth of NPs by the tendency of more metal atoms to aggregate around the existing nuclei and form NPs. Phytochemicals present in plant parts control the nucleation and growth rates, and this subsequently influences NPs’ size and shape [49,50,133]. The change in the solution color is an indication that the necessary NPs have been formed effectively. Palladium, gold, copper, zinc oxide, silver, cobalt, and platinum NPs are created using plant extracts [134]. The mechanism for plant-mediated nanomaterial production is categorized into three stages, including reduction, growth, and termination. The reduction process involves the transfer of electrons from phytoactive substances from metallic ions to nonvalent metal atoms. This includes the formation of a variety of MtNPs from nonvalent metal atoms with a wide range of shapes and sizes, such as rod-like, cubic, triangular, hexagonal, and linear atoms. In the termination phase, the nanomaterials are stabilized by the surrounding medium, which contains phytoactive components that have antioxidant properties [135,136]. Flavonoids, terpenoids, phenolic acids, alkaloids, and proteins are the most common phytochemicals involved in the biosynthesis of NPs and have a significant role in this process [31], as shown in Table 3.

### 5.2. Microbe-Assisted Synthesis

Avoiding harmful, toxic, and hazardous chemicals and high energy consumption required by physicochemical methods, microorganisms play an important role in the synthesis of NPs. For the synthesis of nanomaterials using microbes, fungi, bacteria, microalgae, yeast, viruses, and actinobacteria are the most common microbial agents [146]. Moreover, the rapid growth, simple handling, low cost of cultivation, and ease of genetic modification of microbes are considered key advantages for utilizing microbial routes. This process leads to higher production rates as it is a simple, stable, and robust process [147,148]. Due to naturally detoxifying processes, microorganisms produce inorganic nanomaterials. Intracellular or extracellular redox potentials of these microorganisms have properties such as leakage of enzymes, proteins, and metabolites, and they are essential parameters in the reduction process of metal precursors [103]. Various reductase enzymes help in accumulating and detoxifying metals, therefore reducing metal salts to NPs characterized by minimal polydispersity and a narrow size distribution [149]. The extracellular method for NPs synthesis has gained attraction because it eliminates the downstream steps of processing in the intracellular methods during NPs recovery. In the extracellular method, sonication is used to break down the cell wall; then centrifugation and washing are done for NP purification. During the process of green synthesis, peptides, proteins, organic materials, enzymes, metal-resistant genes, and reducing cofactors act as reducing agents, facilitating NPs synthesis as they are functionalized as natural capping agents, thus having a role in the prevention of aggregation and in stabilizing NPs for a long time [150,151,152,153].

#### 5.2.1. In the Intracellular Synthesis

In the microbial cell, the cell wall is an important component for ion transport. There is an electrostatic interaction through which the negatively charged cell walls trap the positive charge of metal ions. The enzymes found in microbial cell walls are responsible for reducing metal ions and catalyzing their transformation into MtNPs, producing nanoclusters which are then further diffused off by the cell wall into the liquid. Trapping, bio-reduction, and capping are three steps involved in NPs synthesis [154,155,156].

#### 5.2.2. In the Extracellular Synthesis

It involves culturing microorganisms under optimum conditions in a rotating shaker for 1–2 days. Then, centrifugation of the culture is essential to remove the biomass. After that, the supernatant is collected, and metal salt solution is added for NPs synthesis, then incubated once more. The culture medium color will be changed upon synthesis of NPs. Large particles or medium can be removed by centrifugation of the reaction mixture. With density gradient or high-speed centrifugation, followed by washing with ethanol/water, NPs can be collected [43,157]. Accumulation of metal particles either inside or outside the bacterial cells is the initial stage in the synthesis of NPs using microorganisms. The second stage involves the reduction of these metal particles into NPs by enzyme catalysis [41].

### 5.3. Advantages of Green Synthesis Method

Green NPs are characterized by their cost-effectiveness, since the green synthesis process uses less energy and fewer materials [158]. Green NP synthesis offers advantages such as simplicity, affordability, high stability, low time, non-toxic byproducts, and the ability for large-scale production [159]. Some MtNPs are characterized by nontoxicity and biocompatibility in normal cell lines while having high toxicity in cancer cell lines [44]. Biocompatibility features make them suitable for biomedical applications such as diagnostics and drug delivery.

Due to their better attachment to receptors, advanced penetration, and enhanced toxicity, smaller NPs are more effective than larger NPs in targeting cancer cells. The activity and bioavailability of NPs are affected by how efficiently they are internalized by cells and the metal ions that are released thereafter [128]. The cytotoxicity of NPs can be affected by decreasing or increasing their concentration or by removing certain compounds and is based on their solubility, surface area, dose, size, shape, and surface charge. The smaller the NPs’ size, the more enhanced the cellular uptake of NPs. According to many studies, DNA damage and oxidation potential can be enhanced by small-sized NPs < 50 nm [128,160,161,162]. At both the cellular and intracellular levels, green NPs can act upon cancer cells. They possess anticancer properties against different cancer cell types without cytotoxicity [41].

**Figure 2:**
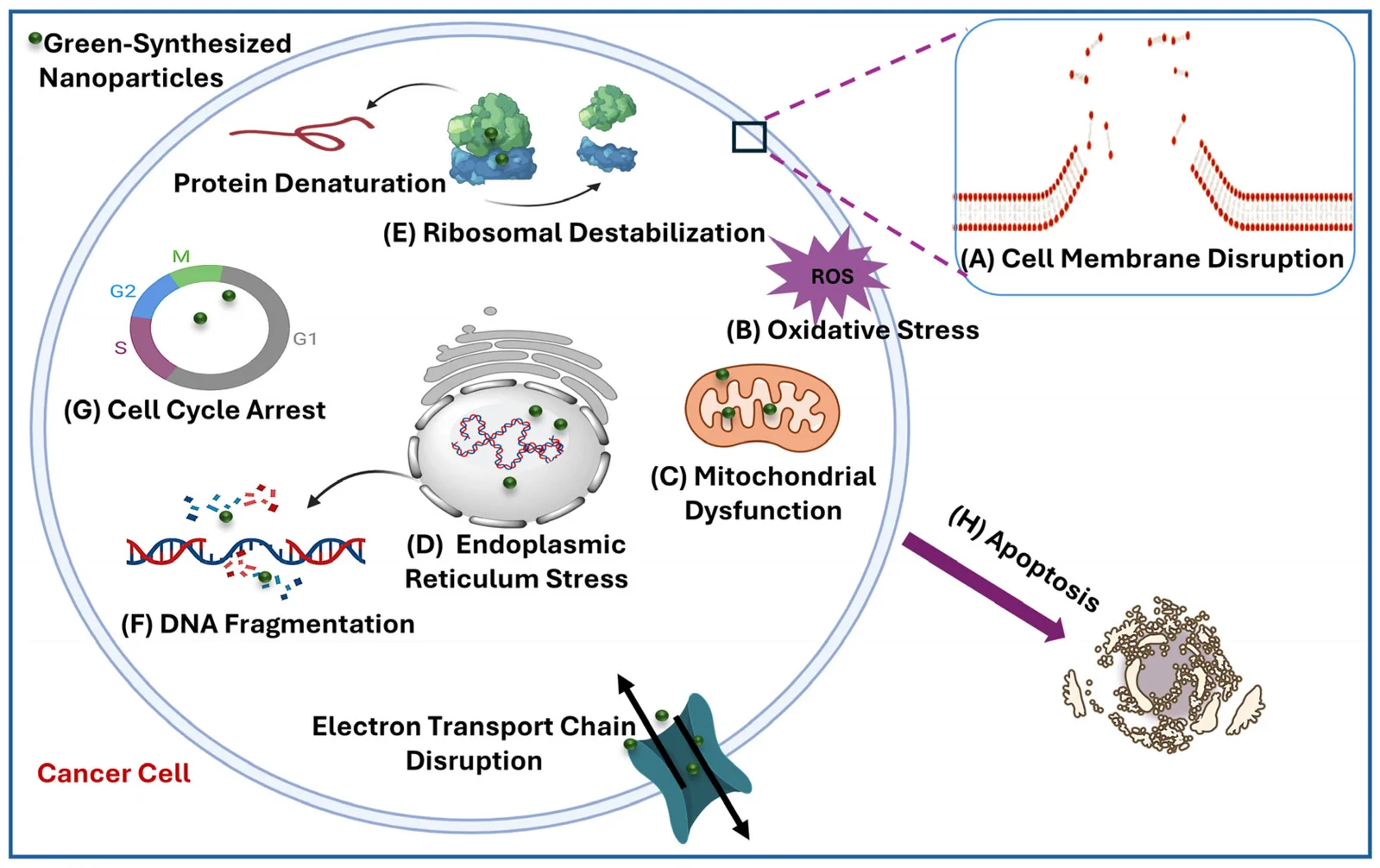
Mechanism of action for green-synthesized NPs as anticancer agents. Following cellular uptake, green-synthesized NPs first interact with the cell membrane (**A**), where they may disrupt membrane integrity and alter membrane permeability. Once internalized, they promote excessive intracellular ROS generation (**B**), leading to oxidative stress. Elevated ROS subsequently impairs mitochondrial function (**C**) by disrupting the mitochondrial membrane potential and electron transport chain, resulting in ATP depletion and activation of apoptotic signaling pathways. Oxidative stress also induces endoplasmic reticulum stress (**D**) together with protein denaturation, enzyme inactivation, and ribosomal destabilization (**E**), thereby impairing essential cellular functions. In parallel, ROS-mediated damage promotes DNA fragmentation (**F**) and cell-cycle arrest (**G**), suppressing cancer cell proliferation and ultimately leading to apoptotic cell death (**H**). In renal cell carcinoma, ROS-mediated mitochondrial dysfunction and apoptosis are among the most frequently reported mechanisms underlying nanoparticle-induced cytotoxicity.

As illustrated in Figure 2, NPs can be uptaken by cancerous cells and can interact with various cellular components such as nucleic acids, lipids, proteins, membranes, enzymes, and organelles due to their physiochemical properties and phytoconstituent content, resulting in molecular and cellular alterations that may be either inhibitory, primarily affecting anticancer activities such as cell viability, growth, tumor invasion, proliferation, and progression, or stimulatory, primarily influencing apoptotic pathways and cell death [163,164]. Efficient cellular uptake and selective cell targeting are advantages of NPs-based therapy. Therapeutic agents are delivered to the interior of cancer cells when ligands or targeting agents bind to their surfaces, triggering endocytosis [164,165]. In addressing cancer therapy development, inducing both intrinsic and extrinsic apoptotic pathways is the predominant mechanism to stop cancer cell proliferation [166]. In vivo studies proved that naturally occurring AgNPs were effective in lung cancer chemoprevention and therapy. It is possible to eradicate cancer cells by activating ROS generation and inducing apoptosis at the subcellular and molecular levels. Research has shown that endophytic fungi-produced AgNPs can kill lung cancer cells (A549) through free radicals scavenging and apoptosis triggering, which is aided by the natural biomolecules integrated into these NPs [128,167].

## 6. Delivery Systems for Kidney Cancer

The kidneys play a vital role in maintaining overall health by producing urine, regulating blood pressure, and balancing electrolytes, water, and acid-base levels. Renal disorders can lead to severe complications, and kidney disease is a growing concern worldwide, creating significant financial strain due to the need for long-term treatment, dialysis, or transplantation [168]. Nanomaterials have emerged as a promising strategy for targeted drug delivery to the kidney [169], as shown in Table 4.

A previous study investigated liposomes for site-specific drug delivery in renal cancer, employing Dal K29-conjugated small unilamellar vesicles encapsulating methotrexate, a monoclonal antibody targeting human renal cancer cells. The study found that these Dal K29-linked SUVs exhibited significantly higher binding to CaKi-1 kidney cancer cells compared to controls. Furthermore, the targeted liposomes demonstrated superior growth inhibition of CaKi-1 cells compared to Dal K29-conjugated methotrexate and unconjugated methotrexate, suggesting the potential of this system for targeted renal cancer treatment [170]. Liposomes, in general, can increase the effectiveness and therapeutic index of drugs, increase drug stability, and reduce the toxicity of encapsulated drugs [171]. Cancer therapy is advancing through the incorporation of nanotechnology, which presents exciting opportunities for improving treatment effectiveness and minimizing side effects. MtNPs, especially those produced via green chemistry from metals like silver, platinum, and gold are leading this innovation [172].

Green-synthesized NPs are nanomaterials generated by eco-friendly methods utilizing biomaterials as reducing and stabilizing agents, including plant extracts, bacteria, or fungi. This environmentally sustainable method employs fewer harmful chemicals and minimizes ecological damage, frequently yielding biocompatible NPs for biomedical applications. Green synthesis fully utilizes the qualities of natural sources, offering a viable, sustainable, cost-effective, and scalable alternative to traditional chemical approaches, particularly in targeted cancer therapy [173]. Conventional cancer therapies, like chemotherapy, are frequently constrained by serious adverse effects and nonselective cell targeting [174]. Green-synthesized MtNPs can be designed with certain physical qualities, like size, shape, and surface features, to more efficiently target cancer cells and reduce the damage to healthy cells [175]. These NPs target cancers by increasing the permeability and retention (EPR) effect, which causes aberrant vascular structures to accumulate preferentially in tumor tissues. Furthermore, these NPs’ ability to bind selectively to cancer cell markers thanks to surface functionalization improves their therapeutic efficacy and selective absorption [169,176]. Photothermal therapy and drug delivery systems both make use of these NPs due to their capabilities, which allow for the controlled release of therapeutic chemicals and localized hyperthermal treatment [177].

Recent advances in green-synthesized NPs have shown promise in improving drug delivery for kidney cancer, offering an eco-friendly and biocompatible alternative to conventional synthesis methods. Plant-derived NPs (e.g., curcumin-loaded AuNPs, quercetin-coated selenium NPs) exhibit enhanced tumor targeting, reduced cytotoxicity, and improved bioavailability compared to chemically synthesized counterparts [178]. These NPs leverage natural phytochemicals as stabilizing and reducing agents, which also contribute to their anticancer effects via ROS generation and apoptosis induction [179]. For instance, green-synthesized AgNPs from *Azadirachta indica* (neem) have demonstrated potent anti-angiogenic effects in RCC by inhibiting VEGF signaling [180]. Similarly, fungal-mediated synthesis of zinc oxide nanoparticles (ZnONPs) has been explored for targeted delivery of mTOR inhibitors like everolimus, minimizing systemic toxicity [181]. However, challenges such as batch-to-batch variability, scalability, and precise tumor homing remain to be addressed. Future research should focus on hybrid green NPs to enhance stability and therapeutic payloads [182]. AuNPs were synthesized using *Curcuma longa* extract, where Curcumin serves dual roles as a reductant and stabilizer [183]. These NPs selectively aggregate in RCC tumors through the EPR effect and induce apoptosis by suppressing NF-κB signaling [184]. Quercetin-Coated Selenium nanoparticles (SeNPs) were prepared using *Allium sativum* (garlic) extract, functionalized with quercetin for targeted delivery [185]. Quercetin enhances cellular uptake, while SeNPs generate ROS, synergistically inhibiting HIF-1α/VEGF pathways in RCC. The result demonstrated 70% higher cytotoxicity in 786-0 RCC cells than conventional selenite [186].

**Table 4:** List of green synthesized NPs and their biological activity as anticancer against RCC.

Nanomaterials	Biological Sources	Shape, Size	Advantages	Reference
Se/Ch	*Streptomyces parvulus* MAR4	Spherical, 94.2 nm; Semispherical, 74.9 nm	SeNPs and Se/Ch-nanoconjugate exhibited cytotoxicity against Caki-1 RCCs with IC_50_ values of 21.35 and 7.83 μg/mL, respectively, indicating greater cellular uptake and potency for the nanoconjugate.	[187]
Pd	*Agaricus bisporus*	Spherical, 13–18 nm	PdNPs exhibited potent cytotoxicity against PK13 RCCs with an IC_50_ of 26.1 μg/mL, as determined by MTT assay.	[188]
Zn	*Agaricus bisporus*	Different shapes, mostly spherical, 12–17 nm	ZnNPs reduced Fe-NTA’s oxidative stress by decreasing lipid peroxidation and increasing antioxidant molecules and demonstrated promising anti-tumor activity by reducing tumor cell infiltration and enhancing apoptosis.	[189]
Au	*Portulaca oleracea*	Hexagonal, 8.8–25 nm	AuNPs exhibited superior activity against UO-31 cells (IC_50_ = 17.47 μg/mL) compared to *P. oleracea* extract (IC_50_ = 29.11 μg/mL).	[190]
Ag	*Malva sylvestris* L.	Spherical, 21.8–79.9 nm	The AgNPs showed cytotoxic activity by MTT assay against HEK293, RCC-GH, RCC-JW (KTCTL-195), and CaKi-2 RCC with IC_50_ values of 147, 135, 118, and 66 µg/mL, respectively.	[68]
Non-metabolizable glucose analog functionalized zinc oxide (ZnO-2DG)	Apple	Sharp petals, 13 nm	ZnO-2DG nanohybrid showed greater inhibition and reduced proliferation against kidney cancer cells compared to ZnONPs and standard drugs (levofloxacin, doxorubicin).	[191]
ZnO	*Holigarna grahamii*	Spherical, 9–16 nm	ZnONPs exhibited anticancer activity against A498 cell line with IC_50_ values of 1.363 and 7.417 μg/mL by MTT and XTT assays, respectively, and induced significant concentration-dependent apoptosis as confirmed by Annexin V and DAPI staining.	[192]
Cu	*Ziziphus ziziphus*	Spherical, 1–100 nm.	The CuNPs showed potent toxicity against A498 cells via triggering an intracellular ROS generation, upregulating Bid, Bax, and caspase (3 and 9), downregulating Bcl-2 gene expression, and inhibiting mTOR/PI3K/AKT protein expression.	[193]
ZnO	*Garcinia cambogia*	Mostly hexagonal, 11–32 nm	ZnONPs exhibited cytotoxicity against human RCCs (A498) that was determined by MTT and XTT assays with IC_50_ values of 1.828 and 18.86 mg/mL, respectively.	[194]
Au	*Curcuma wenyujin*	Spherical, 200 nm	AuNPs induced apoptosis in SW 156 and A498 RCCs (IC_50_ = 40 and 25 μg/mL, respectively), triggered ROS-mediated mitochondrial membrane damage (H2DCFDA), confirmed nuclear damage (Rhodamine 123/DAPI), and modulated apoptotic markers by downregulating Bcl-2/Bcl-xl while upregulating Caspase-3, Caspase-9, Bid, and Bad (qPCR/immunoblotting).	[64]
K1 (ZnO NPs), K2 (La doped ZnO NPs), K3 (Ce doped ZnO NPs) and K4 (Nd doped ZnO NPs)	*Gymnema sylvestre*	K1-K4 samples form a spherical, spindle, hexagonal and flake like nanostructures, size 138, 52, 59, and 63 nm, respectively	K2 showed the highest cytotoxicity against A498 cells (IC_50_ = 35.86 μg/mL) among K1–K4 (41.74, 40.05, and 63.08 μg/mL, respectively).	[195]
Zn/CuO	*Duchesnea indica*	Spherical, 100–300 nm	The synthesized Zn/CuO NPs gave excellent inhibition of 498 kidney tumor cells, which was verified by the histological and CLSM images.	[196]
Y_2_O_3_	*Forsythiae fructus*	Flake-like flower, 11 nm	Y_2_O_3_NPs showed selective cytotoxicity against Caki-2 cells at high concentrations, with no toxicity toward normal MDCK cells (SRB/flow cytometry).	[197]
Fe_2_O_3_	*Psoralea corylifolia* Linn	Spherical, hexagonal, and cubic, 39 nm.	Fe_2_O_3_NPs showed significant cytotoxicity against Caki-2 cells at lower concentrations, accompanied by dose-dependent caspase-3 upregulation (SRB/immunofluorescence).	[198]
Ag	*Carissa carandas*	Spherical, 28–60 nm	The anti-proliferative effect of AgNPs was appraised against the HEK-293 cell line with IC_50_ 22.14 µg/mL by utilizing the MTT test.	[199]
CuO/starch	Starch	Almost spherical, 30–35 nm	CuO/starch nanocomposite exhibited considerable toxicity against human RCC (HEK293, RCC-GH, and CaKi-2) with IC_50_ values of 125, 139, and 208 µg/mL, respectively.	[200]
Sunitinib-loaded micellar nanocomplex (SU-MNC) poly(ethyleneglycol)-conjugated epigallocatechin-3-O-gallate (PEG EGCG)	Green Tea	Spherical, 167.4 nm	SU-MNC improved anticancer efficacy and reduced toxicity in HRCC-xenograft mice versus SU and SU-PM, via synergy between SU and PEG-EGCG carrier.	[201]
Encapsulation of CGA in CNP form (CNP-CGA hybrid)	Chlorogenic acid (CGA)	Irregular spherical, 134.44 nm	CNP-CGA exhibited cytotoxic activity against 786-0 RCC (12 μM; 60.22% viability reduction), with rapid cellular internalization and sustained accumulation over 48 h, attributed to enhanced membrane uptake facilitated by the CNP shell.	[202]
Ag	*Spatoglossum schröederi*	Spherical, 210 nm	AgNPs were tested against several renal cell lines (HEK, VERO, MDCK). The flow cytometry analyses showed that AgNPs induced necrosis of 786–0 cells.	[203]

## 7. Thermal and Photothermal Therapy

**Figure 3:**
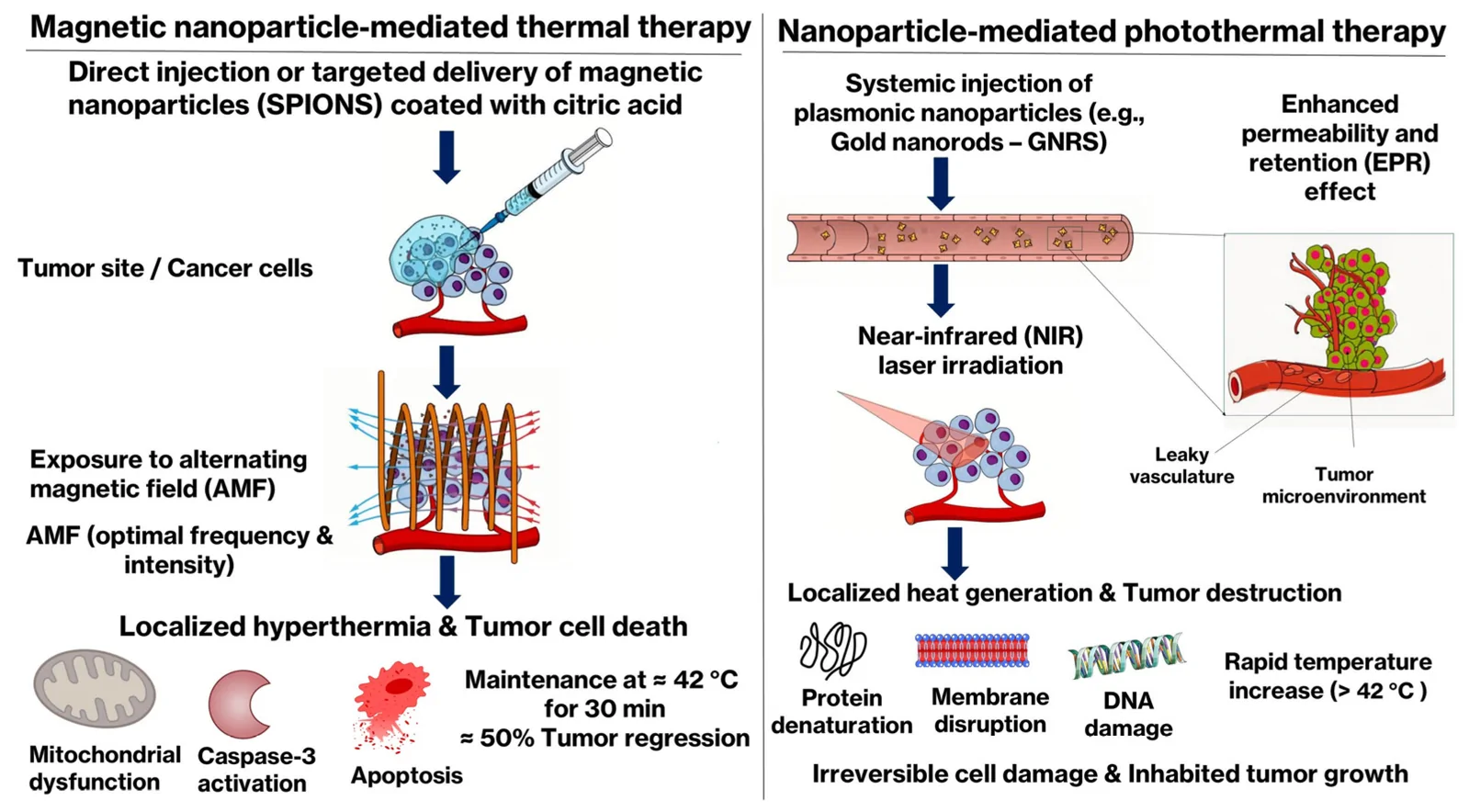
Schematic representation summarizing the distinct mechanisms of NP-mediated thermal and photothermal therapies. Thermal Therapy Mechanism: Magnetic nanoparticles (e.g., citric acid-coated SPIONs) target tumor tissues and are exposed to an AMF. The heat generated induces localized hyperthermia, leading to mitochondrial dysfunction, caspase-3 activation, and cell apoptosis. Photothermal Therapy Mechanism: Plasmonic NPs (e.g., AuNRs) accumulate in the tumor microenvironment via the EPR effect. Upon NIR, laser irradiation generates rapid localized heat (>42°C), inducing protein denaturation, cell membrane disruption, DNA damage, and inhibition of tumor growth.

Over the past twenty years, extensive research efforts in the field of cancer have significantly expanded our understanding of cancer biology. Research has demonstrated that cancer cells possess characteristics such as metastasis, invasion, activation, resistance to growth suppressors, sustained proliferation, evasion of cell death, and the ability to promote angiogenesis. These traits contribute to the complexity and difficulty of cancer treatment [204]. Currently, cancer treatment commonly combines operative procedures, radiation exposure, and drug-based treatments for optimal outcomes. However, a significant limitation of these conventional approaches is their toxicity to healthy cells, leading to numerous adverse side effects for patients [205]. The fundamental mechanisms underlying NP-mediated thermal therapy and PTT are schematically summarized in Figure 3.

### 7.1. Thermal Therapy

Temperature is a crucial factor in biological systems, playing a key role in maintaining viability and ensuring proper functionality. While high body temperatures can negatively impact human health, they can be advantageous for cancer patients. Thermal therapies have played an important role in cancer treatment and were widely utilized during the last quarter of the 20th century. In recent years, interest in these therapies has grown substantially [206].

#### 7.1.1. Thermal Therapy Mechanism of Action

Thermal therapy is a method used for precise and localized temperature regulation. The fundamental concept behind this approach is that applying heat or cold to tissues, including soft tissue, skin, or intra-articular structures, alters their core temperature [207].

The elevation of core tissue temperature causes cellular destruction and structural modifications within the tissue. Despite extensive research on thermal therapy techniques, the precise mechanisms underlying the effects of elevated temperatures on individual cells remain incompletely understood, primarily due to the complexity of host-related factors. In thermal therapy, the targeted tissue is subjected to a controlled temperature increase for a specific duration, which may also induce thermal effects in the adjacent tissues [208]. The therapeutic efficacy of thermal therapy is contingent upon both the duration of heat application and the specific temperature range utilized. This technique involves elevating the temperature of either a localized region or the entire body above normal physiological levels for a controlled period, ranging from a few minutes to several hours, ultimately leading to the destruction of malignant tissue. Based on these parameters, thermal therapies are systematically classified into hyperthermia, diathermy, and thermal ablation [209].

#### 7.1.2. The Role of Magnetic Nanoparticles in Thermal Therapy

Magnetic NPs play a crucial role in thermal therapies. In this approach, these NPs are delivered into tumor cells, either through direct injection or targeted drug delivery mechanisms. Once the magnetic NPs are restricted to the tumor site, they are exposed to an external alternating magnetic field with an optimal frequency and intensity [210].

It causes the magnetic NPs to heat up, which in turn heats the tumor cells, leading to their destruction. The heat generation in magnetic NPs when exposed to alternating magnetic fields is due to mechanisms such as Brownian relaxation, Néel relaxation time, and hysteresis loss. Once the magnetic NPs are heated within the tumor cells, the heat quickly spreads to the surrounding tissues, maintaining a temperature of 42 °C for 30 min, which destroys the tumor cells [211]. Superparamagnetic iron oxide nanoparticles (SPIONs) coated with citric acid were injected into RCC xenografts and exposed to an alternating magnetic field (AMF) (110 kHz, 12 kA/m); this resulted in ~50% tumor regression within 2 weeks [212]. The heat generated disrupts mitochondrial function and induces apoptosis via caspase-3 activation [213].

### 7.2. Nanoparticles in Photothermal Therapy: Mechanism of Action

In PTT, a laser of precise power and wavelength is utilized to induce the destruction of cancer cells. This method involves the exposure of cancer cells, which either contain or are surrounded by NPs, to a specific laser. The interaction between the laser and NPs causes the latter to oscillate, generating localized heat. The laser light excites the electrons within the NPs, which subsequently undergo non-radiative relaxation. This relaxation process results in a rise in the kinetic energy of the NPs, thereby inducing heat generation [214]. The heat generated by NPs is transferred to the surrounding cancerous tissue, causing destruction of cells of cancer [214].

Gold nanorods (AuNRs) hold significant promise as agents for PTT in kidney cancer due to their unique optical characteristics and biocompatibility. The mechanism by which they operate involves several essential mechanisms responsible for the selective killing of cancer cells. After intravenous injection, AuNRs tend to accumulate within tumor tissues. This is largely attributed to the enhanced permeability and retention effect, where the distinctive leaky vasculature of tumors facilitates NP extravasation, coupled with limited lymphatic drainage that causes them to be retained. Once AuNRs are localized in the tumor microenvironment, they are exposed to NIR light, a wavelength that allows for deep penetration into tissues. The interaction between NIR light and AuNRs induces a mechanism known as localized surface plasmon resonance (LSPR). During LSPR, incident light excites electrons on the surface of the nanorods, causing them to oscillate in a collective manner. This collective oscillation generates intense heat, leading to a rapid temperature increase near the AuNRs. The rapid temperature increase (above 42 °C) causes hyperthermia within the tumor tissue, leading to irreversible damage to cancer cells through mechanisms such as protein denaturation, disruption of cell membranes, and DNA damage. Analysis of ultrasound images revealed clear tumor changes, with PTT effectively inhibiting tumor growth. Specifically, real-time temperature control during PTT contributed to this inhibition. Monitoring blood flow showed that tumors treated with AuNRs lacked blood flow, whereas comparative tests still exhibited blood flow. This suggests that the AuNRs treatment induced tumor apoptosis. Apoptosis data confirmed that AuNR-treated tumor cells were largely inactivated in comparison with the control group, demonstrating the effectiveness of PTT [215].

## 8. Gene Therapy and RNA Interference

**Figure 4:**
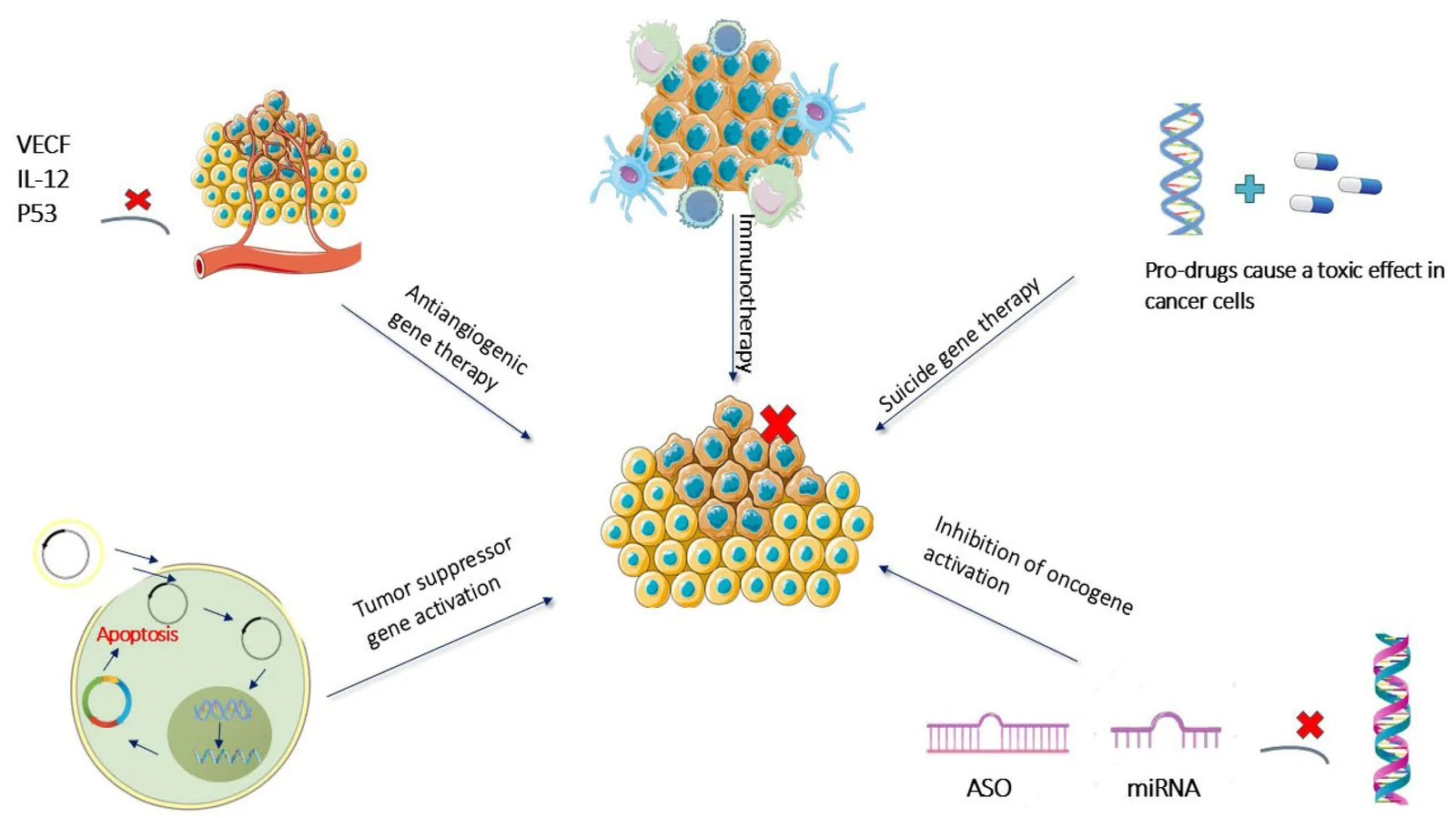
Strategies of gene therapy in cancer treatment are summarized in suicide gene therapy, immunotherapy, inhibition of oncogene activation, activation of tumor suppressor gene, and antiangiogenic gene therapy.

Cancer gene therapy involves the introduction and editing of genetic material. To repair mutant genes, we need to activate the immune system or directly stimulate cancer cell death. Cancer gene therapy contains strategies, including inhibition of oncogene activation, tumor suppressor gene activation, suicide gene therapy, immunotherapy, and antiangiogenic gene therapy [216], as illustrated in Figure 4.

RNA interference (RNAi) is a defense mechanism against external gene invasion. RNAi methods include microRNA (miRNA) and small interfering RNA (siRNA). Targeted gene expression can be reduced in a sequence-specific manner through mRNA translation repression by miRNA or targeted mRNA destruction by siRNA and miRNA [216]. RNAi therapy can be used in cancer treatment, but siRNAs have some challenges owing to their low stability and drug delivery. Unmodified siRNAs are degraded by nuclease enzymes or attacked by the immune system. They are also eliminated by the kidney [217].

NPs can protect nucleic acids from degradation, improve cellular uptake, and target tumor cells specifically; they have emerged as an ideal vehicle for them. mRNA, siRNA and CRISPR components can be delivered using NPs in gene therapy, which enables them to either suppress or repair the genes in cancer therapy [94]. LNPs are used to deliver genome editing tools. One potential DNA carrier, namely plasmids, is a lipid-like structure that can be broken down by disulfide bonds (SS). LNPssPalmE—LNPs incorporating plasmid DNA (pDNA)—was created to overcome problems with pDNA and achieve targeted gene expression for anti-angiogenic objectives in RCC [218]. LNPs are used to deliver siRNA. ALN-VSP is an RNAi therapeutic method developed by Alnylam Pharmaceuticals and formulated as LNPs containing siRNAs targeting kinesin spindle protein (KSP) and VEGF. Through RNAi-mediated gene silencing, ALN-VSP reduces the expression of KSP and VEGF in patients who have serious solid tumors. Silencing the gene for P-glycoprotein, a protein that removes numerous foreign compounds from cells and functions as a drug exporter, is another advantage of molecular targeted therapy that may help manage the issue of multi-drug resistance. The cytotoxicity of chemotherapeutic drugs against cancer cell lines can be increased by creating a nanosystem that uses mesoporous silica NPs to deliver siRNA which suppresses the expression of P-glycoprotein genes and act as an anti-cancer agent simultaneously [219]. There is a study demonstrating an NP-based delivery system (PSPM-NPs) made of polydopamine (PDA) NPs, liposomes, and MUC12 antibodies to explore the role of lncRNA-SLERCC as a tumor suppressor in RCC. Through the alteration of the Wnt/β-catenin signaling pathway, this targeted delivery system efficiently inhibits tumor progression and metastasis by facilitating the transfer of the SLERCC plasmid into RCC cells [220].

Mesoporous silica NPs (MSNs) specially designed NPs successfully target cancer cells and have a high nucleic acid loading capacity, allowing for endosomal escape and regulated release. The special surface characteristics of MSNs make it easier to design and decorate them for endosomal escape, immune system evasion, tumor cell targeting, regulated cargo release, and high nucleic acid loading [221]. Positively charged skeleton dendrimers, such as PAMAM, PPL, and PLL, are appropriate nonviral vectors for delivering genes. Through electrostatic interaction, their positive charge enables them to form a stable complex with DNA that can pass through cell membranes and enter the nucleus [222]. The efficient gene transport vector must prevent intracellular lysosomal degradation while shielding the foreign DNA from degradation and deactivation. CNTs have garnered a lot of interest in this context as non-viral gene carriers. Via a variety of mechanisms, CNTs interact with several biological components to facilitate effective cellular uptake in diverse cell types, exhibiting more immunogenic and fewer immunogenic side effects on the cells [223].

## 9. Challenges and Limitations

Green-synthesized NPs offer several advantages over their chemically synthesized counterparts, particularly in biomedical and anticancer applications. Green synthesis utilizes plant-, microbial-, or biomolecule-derived reducing and stabilizing agents, resulting in NPs with enhanced biocompatibility and reduced dependence on hazardous chemicals. They have demonstrated comparable or improved anticancer efficacy, partly due to the presence of bioactive surface phytochemicals that may enhance cellular uptake, ROS generation, and tumor cell apoptosis. Moreover, these NPs often exhibit lower toxicity toward normal cells and improved safety profiles compared with chemically synthesized analogs. However, despite these promising findings, direct comparative data on pharmacokinetics, biodistribution, and long-term toxicological outcomes remain limited. In contrast, chemically synthesized NPs generally provide superior control over particle size, morphology, and batch-to-batch reproducibility, facilitating scalability and regulatory standardization. Nevertheless, the use of toxic reagents and the generation of hazardous byproducts remain significant drawbacks. Therefore, while green synthesis represents a more sustainable and potentially safer approach for developing anticancer nanomedicines, challenges related to particle uniformity, large-scale manufacturing, and regulatory readiness must be addressed before widespread clinical translation can be achieved [158,224].

The green synthesis of nanomaterials is relatively simple and has several potential applications, especially in biological contexts; however, several restrictions and issues need to be resolved, including toxicity concerns, regulatory hurdles, scalability, and reproducibility. Firstly, the role of phytochemicals in the green extract can be concluded, while the elucidation of the specific reaction mechanisms between biological compounds and nanomaterials represents a challenge during the synthesis process. Additionally, the reactivity of the nanomaterials in biological systems is changed by the alteration of the phytochemicals’ function as capping and/or reducing agents. For example, the root extract of *Sageretia thea* acts as a strong reducing, capping, and stabilizing agent for the synthesis of IONPs, which have several biological activities such as enzyme inhibition, cytotoxicity, antioxidant, and antimicrobial activities [225]. The *Curcuma longa* L. flower extract acts as a reducing and capping agent for synthesized AgNPs with antibacterial properties [226]. Therefore, there is a demand for comprehensive toxicity and biocompatibility studies, especially in vivo studies, which are essential for understanding the long-term impact on health and the environment.

Secondly, scalable and repeatable synthesis techniques for eco-friendly NPs production are another challenge. The key considerations for scaling up manufacturing include variation in raw materials, optimization of reaction conditions, and standardization of synthesis techniques. These parameters affect the properties of NPs, leading to batch-to-batch variability. The selection of suitable biomaterials is essential since not all sources are suitable for scaling up the formulation and production of consistent characteristic NPs. Different plant parts, seasons, or microbial strains and growth circumstances contribute to the variability in biological sources. Furthermore, time constraints hinder the use of raw biomaterials in worldwide production due to the specific season of collection, and each season varies widely in different regions. Therefore, the sustainable production of green synthesized NPs is possible locally, although it is challenging globally [227]. On the other hand, microbial synthesis is devoid of such drawbacks, but it requires many precautions. The maintenance of culture conditions and a sterilized environment is among these requirements, which may be relatively complex [228].

The lack of standardized synthesis parameters, including reaction temperature, pH, reaction time, and the concentrations of the plant extract and metal salt precursor, makes batch-to-batch reproducibility a major challenge. For example, the mixed solution of *Quercus incana* leaf extract and 1 mM silver nitrate (AgNO_3_) needs to be kept at 25 °C overnight [229]. The aqueous *Carissa carandas* leaf extract was mixed with 1 mM AgNO_3_ at room temperature for 60 min [230]. The mixture of *Cucumis prophetarum* and 1 mM aqueous AgNO_3_ was incubated at 80 °C for 3 h with constant stirring [231]. Aqueous *Moringa oleifera* leaf extract mixed with 1 mM AgNO_3_ was kept in the dark for five days until the color stabilized [232].

The size and shape of NPs vary widely due to the involvement of diverse extract phytochemicals like phenols, flavonoids, and terpenoids during the synthesis process. Many reports show quite a broad range in particle size and different shapes of the synthesized NPs, which makes controlling NP quality a major challenge. The particle size of CuNPs synthesized from *Ziziphus zizyphus* leaves ranged from 1 to 100 nm [116], while the AgNPs synthesized from pineapple leaf waste varied from 40 to 150 nm [233]. The particle size of AuNPs synthesized from *Abies spectabilis* extract also differed greatly, wherein it ranged from 20 to 200 nm [234]. On the other hand, the synthesized AuNPs using *Gelidiella acerosa* aqueous extract formed numerous shapes of spherical, hexagonal, and cubical NPs with aggregation [235], and the ZnNPs synthesized by white mushroom *Agaricus bisporus* also have different shapes, mostly spherical [112]. AgNPs synthesized by *Chlorella ellipsoidea* also had other shapes, such as cubic, triangular, and rod [225]. Standardization of synthesis protocols and quality control measures emphasizes the importance of achieving consistent NPs quality and optimal performance in biomedical applications across multiple production batches, which is essential for reproducibility and reliability in-scale manufacturing. Finally, the regulatory hurdles are associated with all the above-mentioned challenges. The regulatory agencies, like the FDA, EMA, and EPA, are responsible for the approval and registration of NP-based products for pharmaceutical and environmental applications. Thus, the detailed descriptions of nanomaterial safety, efficacy, identity, purity, and reproducibility are requirements for regulatory approval and market acceptance [236].

## 10. Future Directions

Green-synthesized NPs are attracting growing interest as potential therapies for kidney diseases. However, moving these materials from experimental studies to clinical use remains difficult. We propose a future research framework comprising three sequential phases including preparation, validation, and translation.

A logical starting point is the standardization of the biological source itself, whether plant-, microbial-, or waste-derived. In plant-mediated synthesis, variability can be reduced by documenting cultivation conditions, including climate, soil characteristics, and harvest season, while maintaining consistent extraction procedures. Monitoring major bioactive constituents through techniques such as LC–MS and NMR may further support batch-to-batch consistency [237]. When microorganisms are employed, priority should be given to strain authentication through 16S rRNA or ITS sequencing and the maintenance of master and working cell banks [238]. Controlling factors such as medium composition, incubation conditions, and harvest stage may help limit variations that affect NPs formation. In addition, several considerations apply to agricultural and food waste-derived materials, as their composition is often influenced by factors such as origin, storage environment, and pre-treatment methods. Tracking processing history and measuring bioactive components could enhance the reliability of nanoparticle production from these inherently heterogeneous resources [239].

Once the biological source material is standardized, the synthesis process itself must move from empirical trial-and-error to statistically controlled methods. Traditional one-variable-at-a-time approaches cannot capture interactions among multiple parameters, such as pH, temperature, precursor concentration, biological source concentration, and reaction time. In contrast, design of experiments (DoE) and response surface methodology (RSM) enable multidimensional optimization with fewer experiments and identify the parameter window that minimizes variance in NP size and polydispersity [240,241]. To maintain optimal synthesis conditions throughout the reaction, future systems should incorporate closed-loop control with real-time sensors. Autonomous systems using machine learning can self-adjust parameters without human intervention, continuously monitoring pH, temperature, redox potential, and optical density to maintain optimal synthesis conditions throughout the reaction [242].

Batch release testing should be established as a routine practice for all biological sources to ensure successful clinical translation. Every batch intended for in vivo studies should be characterized using a core analytical panel, as summarized in Table 5. Establishing well-defined acceptance criteria is equally important, as it enables objective pass/fail decisions before proceeding to animal studies. For kidney-targeted nanocarriers, acceptance criteria should be linked to the intended site of action, with size thresholds and surface properties defined according to the target renal compartment.

The integration of artificial intelligence (AI) into green NPs synthesis is receiving increasing attention as a tool to improve process optimization and reproducibility. Machine learning (ML) and deep learning (DL) approaches can help identify suitable synthesis conditions, including pH, temperature, precursor concentration, and biological source composition, thereby reducing experimental effort. Models trained on experimental datasets may also predict NP properties from synthesis inputs derived from plant extracts, microbial cultures, or waste-based materials [243,244]. For renal applications, AI-based predictive models may assist in evaluating nephrotoxicity and other safety-related endpoints, supporting early-stage risk assessment during NP development [245].

Once reproducible NPs are prepared, their behavior in biological environments must be rigorously validated before any claim of renal targeting can be made. This phase focuses on NP stability in biological fluids, renal clearance and biodistribution, intra-renal localization, and long-term safety, which together represent the key components of preclinical evaluation for renal-targeted nanomedicines.

Stability assessments remain underreported in many studies involving green-synthesized NPs. This limitation is particularly relevant for formulations intended for intravenous administration and renal targeting, where changes in NPs stability may influence biodistribution, circulation time, and kidney accumulation. Future studies should therefore incorporate a standard panel of stability assessments, including protein corona formation, colloidal stability in physiological media, aggregation behavior, and freeze–thaw stability.

Protein corona analysis represents an important first step, as adsorption of serum proteins onto the NP surface can alter physicochemical properties and affect biological performance. Changes in hydrodynamic diameter and surface charge following exposure to biologically relevant media may provide insight into the likelihood of corona formation and its potential impact on renal clearance [246,247]. In parallel, colloidal stability should be evaluated under physiological conditions, including buffer solutions, cell culture media, and serum-containing environments, to determine whether particle aggregation occurs over time. Aggregation studies may provide additional information regarding NP behavior during storage and biological exposure. Monitoring particle size distribution using techniques such as DLS and UV–Vis spectroscopy can help identify gradual changes that may not be detected during short-term assessments [248]. Determination of the critical aggregation concentration (CAC) may also assist in defining concentration ranges that maintain colloidal stability. For formulations intended for long-term storage, stability during storage, reconstitution, and freeze–thaw processing should be evaluated to ensure that NP physicochemical properties remain unchanged during handling and storage [249]. A widely used technique for quantifying renal clearance and biodistribution is ICP-MS, which measures metal concentrations with high sensitivity. Using metal-based NPs as model systems, urine collected over multiple time points can be analyzed to determine the fraction excreted renally, while the remainder is either retained in tissues or cleared hepatobiliary [250,251]. The same analytical platform can be used to quantify NP accumulation in major organs (kidney, liver, spleen, lung, heart, brain) over extended periods, providing a complete mass balance. Such studies also inform chronic toxicity, as slowly accumulating NPs may show little short-term effect but significant long-term toxicity [252].

Within the kidney itself, localization matters enormously. The kidney contains functionally distinct compartments. including glomeruli and proximal tubules. Using TEM or immunofluorescence with kidney-specific markers, researchers can determine whether NPs accumulate in glomerular cells or in proximal tubular epithelial cells. This distinction is critical because different renal diseases affect different compartments. Diabetic nephropathy primarily affects glomeruli, while acute kidney injury from cisplatin or sepsis primarily damages proximal tubules. An NP that accumulates in glomeruli suits glomerular diseases but is less effective for tubular diseases—and vice versa [253]. The literature has established reasonably clear size-dependent localization patterns. For rigid NPs, such as AuNPs and quantum dots, the glomerular filtration cutoff is generally reported to be around 6–8 nm [254,255]. For soft or deformable NPs, including polymers and dendrimers, the effective filtration threshold may be larger because their flexibility can facilitate passage through filtration barriers [256]. Shape also influences renal clearance; for example, high-aspect-ratio nanomaterials such as SWCNTs can undergo renal filtration despite their extended length because their small diameter governs transport through the glomerular barrier [257]. NPs near the glomerular filtration threshold may undergo filtration followed by tubular interactions, including megalin-mediated uptake by proximal tubular cells, making this design concept relevant for tubular targeting strategies. In contrast, larger NPs, particularly those in the 75–100 nm range, have been reported to preferentially localize within the mesangial niche, making them suitable for glomerular or mesangial disease targeting [258].

For researchers developing green NPs to treat RCC, the choice of preclinical model is critically important. The most common model, the subcutaneous xenograft, in which human cancer cells are implanted under the skin of immunodeficient mice, has important limitations for renal cancer studies. Subcutaneous tumors grow in a location with completely different vascularization, interstitial fluid pressure, and immune cell infiltration compared to tumors growing in the kidney parenchyma [259]. Consequently, subcutaneous models systematically overestimate the therapeutic efficacy of nanomedicines. The preferred alternative is the orthotopic model, where cancer cells are implanted directly into the kidney. For mouse RCC studies, the RENCA cell line injected into the left renal subcapsular space of immunocompetent BALB/c mice is the most established model [240]. The surgical procedure is well-documented, and tumor growth can be monitored non-invasively using imaging techniques such as ultrasound, micro-CT, or MRI [260,261]. The choice between syngeneic and xenograft models depends on the research question. Syngeneic models (RENCA in BALB/c mice) have intact immune systems, making them suitable for studying immunomodulation and combination therapies. Xenograft models (human RCC cells such as 786-0 in immunodeficient Nude mice) allow testing of human-specific drugs but provide no information about immune responses. For more personalized approaches, patient-derived xenograft (PDX) models where a fresh human tumor fragment is implanted into an immunodeficient mouse better preserve the genetic and histological complexity of the original tumor, although they are more expensive and variable [262,263]. Nevertheless, the therapeutic efficacy observed in the subcutaneous model should ultimately be validated in an orthotopic model before any claim of clinical relevance is made. Many promising nanomedicines have failed in clinical trials precisely because they were optimized against subcutaneous tumors that did not recapitulate the human disease.

Beyond localization, chronic toxicity assessment is essential for clinical translation. Histopathological evaluation of kidney tissue using standard stains (e.g., H&E for general pathology, Masson’s trichrome for fibrosis) can detect tubular necrosis, glomerular sclerosis, inflammatory cell infiltration, and interstitial fibrosis [264]. Such findings warrant careful risk–benefit assessment before further clinical development. Finally, mass balance studies differentiating urinary from fecal excretion help determine the relative contribution of renal versus hepatobiliary clearance. NPs mostly excreted in feces may have limited kidney access, whereas one appearing in both urine and feces has a mixed clearance profile. NPs retained in the body with minimal excretion in either route may indicate higher long-term toxicity risk [265].

The final phase addresses how green NPs compare to existing nanomedicines and what regulatory hurdles must be overcome before they can enter human trials.

While direct therapeutic comparisons between green NPs developed for renal diseases and FDA-approved nanomedicines are not appropriate because of differences in disease indications, target tissues, and mechanisms of action, approved nanomedicine products still provide valuable translational benchmarks [266]. Several FDA-approved nanomedicines, including Doxil^®^, Abraxane^®^, Onpattro^®^, and Vyxeos^®^, illustrate the regulatory and manufacturing standards required for successful clinical translation [267]. These standards are closely aligned with the quality benchmarks discussed for green NPs, including synthesis reproducibility, physicochemical characterization, pharmacokinetic profiling, safety evaluation, and batch release criteria. Despite progress in addressing these parameters, an important challenge that remains largely unexplored is the economic feasibility of large-scale production [268]. Future studies should therefore evaluate manufacturing costs, process scalability, raw material availability, and production robustness to determine whether green NP platforms can be translated into commercially viable nanomedicines.

A major challenge for the clinical translation of green NPs is their regulatory classification. Unlike conventional nanomedicines, which are typically manufactured from well-defined synthetic materials, green NPs contain biologically derived surface components that may complicate product characterization and regulatory approval. Although regulatory agencies have established guidance for both nanomedicines and botanical products, green-synthesized NPs do not fit neatly into either category and may require hybrid evaluation pathways. From a manufacturing perspective, production must comply with current Good Manufacturing Practice (cGMP) requirements, including validated processes, quality-control procedures, and batch-to-batch consistency [266]. In addition, regulatory evaluation may be complicated by the principles outlined in the International Council for Harmonisation (ICH) Q11 guideline, which requires clear identification of the active pharmaceutical ingredient (API) [269]. For green NPs, it remains unclear whether the API should be defined as the NP core, the bioactive capping layer, or the NP as an integrated system. Addressing these regulatory and manufacturing challenges will be essential for the successful clinical translation of green nanomedicines.

In summary, we propose that many of these regulatory challenges can be addressed through the standardization and quality-control strategies outlined in the earlier phases of this framework. Establishing consistent manufacturing processes, clearly defined quality attributes, and robust batch-to-batch reproducibility will be critical for facilitating regulatory approval and clinical translation of green nanomedicines.

## 11. Conclusions

Kidney cancers pose a significant clinical challenge, necessitating effective and safe therapeutic approaches. Conventional treatments include surgical resection, ablation, radiation, immune checkpoint inhibitors, tyrosine kinase inhibitors, and chemotherapy, which provide kidney removal or tumor growth tracking over time but are often associated with adverse effects such as high prices, tumor recurrence, and drug resistance. The systematic management of kidney cancers, including therapy, has been greatly advanced by the numerous nanomedicines developed via extensive research and clinical trials. As an alternative, nanotechnology has shown promising drug delivery and therapeutic properties for kidney cancer. Various organisms, such as fungi, bacteria, actinomycetes, yeast, and plants, are used to produce biologically mediated NPs. These findings highlight that the incorporation of natural extracts during the synthesis process brings a growing demand for safe and environmentally friendly aspects to the possible uses of NPs in cancer treatment. Studies focusing on the characteristics of biosynthesized NPs as effective agents for treating cancer based on their biocompatibility and non-toxicity with normal cells, as well as cytotoxicity against cancer cells, are needed.

## Figures and Tables

**Table 1:** Side effects of traditional drugs used for RCC.

Drug	Mode of Action	Side Effect	Reference
Cisplatin, 5-Fluorouracil, doxorubicin, and gemcitabine	Target unlimited and rapidly dividing cells.	Hair loss, appetite loss, vomiting, nausea, diarrhea, fatigue, mouth sores, and bleeding	[64]
Anti-programmed cell death protein, such as nivolumab, pembrolizumab, avelumab, and anti-cytotoxic T-lymphocyte associated protein 4, such as ipilimumab	Prevent cancer cells from expressing certain proteins by targeting them. The immune system acts by recognizing cancer cells and destroying them. The first two drugs act by inhibiting programmed death protein 1 (PD-1). The third drug is known as a PD-1 inhibitor, as it acts by blocking its ligand. The fourth and last drug acts by inactivating T cells, as its action is by CTLA-4 inhibition.	Colitis, rash, pruritus, endocrinopathy, and fatigue	[19,65,66]
Everolimus, vistusertib and temsirolimus	Directly address the molecular drivers of RCC through serine–threonine kinases that control the multiplication of cells by inhibiting mammalian target of rapamycin and regulating the cell cycle.	Hyperglycemia, anemia, edema, diarrhea, hyperlipidemia, metabolic issues, stomatitis, fatigue, rash, and thrombocytopenia	[65,66]
Bevacizumab, axitinib, cabozantinib, lenvatinib, pazopanib, sorafenib, sunitinib and tivozanib	Multikinase inhibitors mainly inhibit receptors of protein kinases such as vascular endothelial growth factor receptor (VEGFR).	Hypothyroidism, myelosuppression, stomatitis, proteinuria, exhaustion, hand-foot syndrome, diarrhea, nausea, hypertension, and fatigue	[67]
Interleukin-2 and Interferon-α	Enhancing anti-cancer activity, they are used to treat cancer by regulating the immune system’s behavior, as they can regulate activation, proliferation, and differentiation of different cell types.	Flu-like symptoms, fatigue, low blood pressure and organ toxicities	[34]

**Table 2:** Comparative features of nanocarriers for kidney cancer drug delivery.

Feature	Liposomes	Dendrimers	Micelles
Drug loading capacity	High (hydrophilic and hydrophobic drugs)	High due to branched architecture and multiple functional groups	High for hydrophobic drugs
Surface modification	Good	Excellent	Good
Renal targeting potential	Can be enhanced using targeting ligands	Can be functionalized for kidney-specific delivery	PAMs have shown megalin-mediated kidney targeting
Systemic clearance	Generally prolonged circulation; depends on PEGylation and size	Size- and generation-dependent clearance	Often rapid unless stabilized or modified
Main advantage	Clinically established platform	High loading and multifunctionality	Stimuli-responsive and kidney-targeting potential
Main limitation	Stability and drug leakage	Potential toxicity at higher generations	Lower structural stability

**Table 3:** Plant phytochemicals and their mode of action.

Phytochemicals	Definition	Mode of Action in the Biosynthesis of NPs	Effect on NPs Properties	Reference
Flavonoids (quercetin and catechins)	They are known for their polyphenolic compounds that are abundant in different fruits, vegetables, and herbs and have antioxidant activity.	- The multiple hydroxyl (-OH) groups of flavonoids enhance reduction of metal ions and formation of flavonoid-metal ion complexes that promote transfer of electrons. - They increase the biocompatibility of NPs and reduce toxicity.	- Influence morphology, size, and stability. - Increase NPs’ biocompatibility and reduce toxicity.	[137,138]
Phenolic acids (caffeic acid and gallic acid)	A class of phenolic compounds that utilize their (OH) groups to reduce metal ions.	- They form stable complexes with metal ions, so they act to reduce them and stabilize NPs. - Prevent aggregation by capping the NPs.	Enhance the antioxidant activity of NPs.	[139]
Terpenoids (geraniol and limonene)	They are organic compounds known as isoprenoids in which hydrocarbon structure and functional groups interact with metal ions to facilitate NPs synthesis.	- They reduce metal ions by electron donation. - They form a protective layer around NPs to stabilize them.	Influence NPs dispersion, biocompatibility, drug loading capacity, and hydrophobicity	[140,141]
Alkaloids (nicotine and caffeine)	They are naturally occurring compounds characterized by the presence of basic nitrogen atoms and are recognized for their pharmacological properties.	- Through their nitrogen-containing structures, they donate electrons to reduce metal ions and facilitate their conversion to NPs. - They have antimicrobial properties that enhance NPs’ applications in biomedical fields.	- Influence the morphology of NPs. - Improve antimicrobial properties.	[142,143]
Proteins (plant enzymes)	Polymers of a large chain of amino acids, such as peroxidases and catalases, plant enzymes enhance NPs synthesis.	- Provide a catalytic effect. - NPs stabilization via capping mechanism. - Prevent agglomeration by forming a protective layer. - Provide steric hindrance and electrostatic repulsion by their unique structure to stabilize NPs.	Enhance properties of NPs for targeted applications.	[144,145]

**Table 5:** Recommended tests for batch release of green-synthesized NPs.

Technique	Purpose
TEM	Determine NP size, shape, and morphology
DLS	Evaluate particle size distribution and colloidal uniformity
Zeta potential analysis	Assess colloidal stability and surface properties
FTIR	Identify biomolecules and surface functionalization
XRD	Determine crystal structure and phase composition
AAS	Quantify metal content and residual metal ions
Limulus Amebocyte Lysate (LAL) assay	Detect endotoxin contamination originating from associated Gram-negative bacteria
DNA/protein assays	Evaluate residual DNA, proteins, or other biological contaminants depending on the purification process

## Data Availability

No new data were created or analyzed in this study. Data sharing is not applicable to this article.
